# Prepregnancy overweight and obesity and long-term risk of venous thromboembolism in women

**DOI:** 10.1038/s41598-023-41186-2

**Published:** 2023-09-05

**Authors:** Ahmad Mahmoud, Katarina Glise Sandblad, Christina E. Lundberg, Gustaf Hellsén, Per Olof Hansson, Martin Adiels, Annika Rosengren

**Affiliations:** 1 Department of Molecular and Clinical Medicine, Institute of Medicine, Sahlgrenska Academy, University of Gothenburg, Gothenburg, Sweden; 2 Department of Medicine, Geriatrics and Emergency Medicine, Region Västra Götaland, Sahlgrenska University Hospital/Östra, Gothenburg, Sweden; 3 Department of Food and Nutrition, and Sport Science, University of Gothenburg, Gothenburg, Sweden; 4 School of Public Health and Community Medicine, Institute of Medicine, Sahlgrenska Academy, University of Gothenburg, Gothenburg, Sweden

**Keywords:** Epidemiology, Health care

## Abstract

Overweight and obesity rates have increased in recent decades, particularly among the younger population. The long-term consequences of obesity with respect to early venous thromboembolism (VTE) in women have not been established. The aim was to investigate the association between body mass index (BMI) in early pregnancy as a proxy for non-pregnant weight and long-term post-pregnancy risk of VTE in women. This registry-based prospective cohort study analysed data from the Swedish Medical Birth Registry, linked to the National Patient and the National Cause of Death Registries for information on post-pregnancy long-term risk of VTE. Cox proportional hazards model were used to determine the association between BMI at baseline and VTE events during follow-up starting 1 year after baseline. The mean age at registration was 27.5 (standard deviation, 4.9) years. During a median follow-up duration of 12 years (interquartile range, 6–21 years) starting 1 year after the first antenatal visit, 1765 and 2549 women had a deep vein thrombosis and/or pulmonary embolism. The risk of VTE linearly increased with increasing BMI. Compared to women with 20 ≤ BMI < 22.5 kg/m^2^, women with high normal weight, i.e. with a BMI of 22.5–25.0 kg/m^2^, had an adjusted hazard ratio (HR) of 1.30 (95% confidence interval [CI] 1.19–1.41), whereas those with a BMI of 30–35 kg/m^2^ and ≥ 35 kg/m^2^ (severe obesity) had an adjusted HR of 2.35 (95% CI 2.04–2.70) and 3.47 (95% CI 2.82–4.25, respectively. Using BMI in early pregnancy as a proxy for pre-pregnancy or non-pregnant BMI in young women, we found a significantly increased risk of post-pregnancy long-term risk of VTE even in those with high normal BMI, compared with lean women, whereas those with severe obesity had a markedly high risk.

## Introduction

Venous thromboembolism (VTE), which includes deep vein thrombosis (DVT) and pulmonary embolism (PE), is the third most common manifestation of acute cardiovascular disease, after myocardial infarction and stroke^1^. Approximately 10 million cases of VTE are reported to occur annually worldwide, with an annual incidence of 39–115 and 53–162 per 100,000 population for DVT and PE, respectively^2^. With increasing life expectancy, the number of patients with VTE is expected to increase, and the annual incidence rates may potentially increase to 700 per 100,000 in individuals aged > 70 years^3^.

The prevalence of overweight, obesity and severe obesity has markedly increased over the last decades, especially among young individuals^4^. The World Health Organization (WHO) defines underweight as body mass index (BMI) of < 18.5, normal weight as 18.5–24.9, overweight as 25–25.9, obesity as > 30 and severe obesity as > 35.0 kg/m^2^. According to the WHO, the worldwide prevalence of obesity tripled between 1975 and 2016, with 13% of all adults estimated to be obese in 2016 (15% in women and 11% in men)^5^.

Overweight and obesity are well-known risk factors for VTE^6, 7^; however, the 2019 European Society of Cardiology (ESC) Guidelines on VTE considered obesity as a weak risk factor^1^. Furthermore, most of the cited literature was either dated, included only a limited number of cases, or did not address the risks associated with the highest BMI groups^8, 9^. Conversely, a recent large-scale study using individual data from 75 predominantly middle-aged population cohorts with > 700,000 participants found that BMI was a strong risk factor for VTE, which was stronger for PE than for DVT^10^. A strong relationship between overweight and obesity in men during young adulthood and the risk of midlife VTE has also been shown^11^; however, to which extent this applies to young women has not been established. Given the comparative rarity of VTE in younger women large populations are needed. The Medical Birth registry (MBR) in Sweden with a very high coverage registers weight and height in virtually all women in Sweden who give birth. We used data on BMI assessed in early pregnancy in women and subsequent risk of VTE.

## Materials and methods

### Study population and design

**Figure 1:**
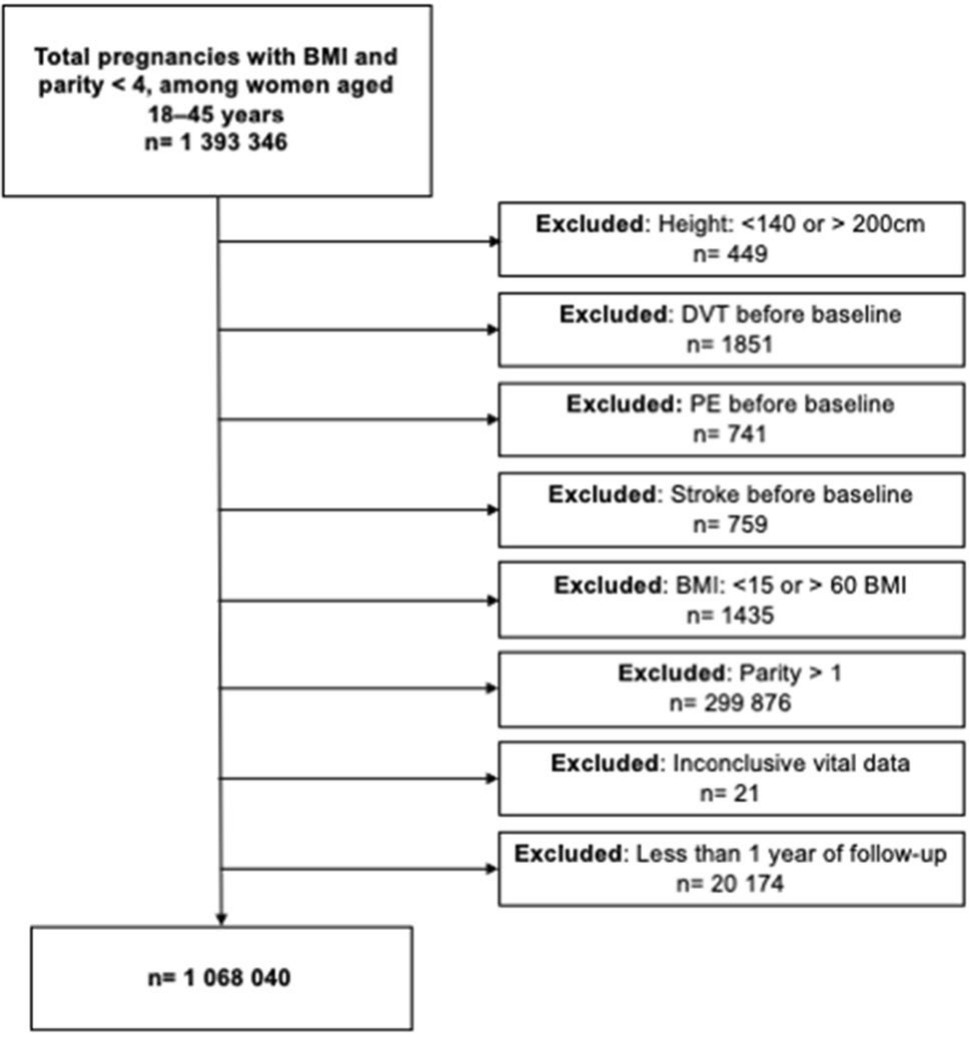
Flow chart of the study population with reasons for exclusion and final study population. *MBR* medical birth registry, *DVT* deep vein thrombosis, *PE* pulmonary embolism, *BMI* body mass index.

The MBR was used to identify all women in Sweden aged between 18 and 45 years who gave birth to their first child between January 1, 1982, and December 31, 2014. Women were included in the study at the date of their first antenatal visit, usually between 6 and 12 weeks of gestation (90% of women). Women who were first registered with their second child (having given birth abroad or before the start of the registry) were excluded, as were women with BMI < 15 or > 60, inconclusive vital data, those with improbable data on weight and height, women with history of VTE or stroke, and those with < 1 year of follow-up (Fig. 1).

### Ethical considerations

The study procedure was approved by the Ethical Committees at the Sahlgrenska Academy at the University of Gothenburg (Dnr:103-15). All personal data were replaced by a code when analyzed (pseudonymized) and therefore informed consent was waived by Ethical Committees at the Sahlgrenska Academy at the University of Gothenburg. The investigation conforms with the principles outlined in the Declaration of Helsinki. All methods were carried out in accordance with relevant guidelines and regulations.

### Data sources

Clinical data were extracted from the Swedish MBR and maintained by the Swedish Board of Health and Welfare. The Swedish MBR compiles information on peri- and antenatal factors and their importance for the health of the mother and infant, including data on all pregnancies that resulted in births in Sweden from 1973 and onwards, with a coverage of approximately 99%. Body weight has been registered since 1982 and self-reported height since 1992. Data on height for the period of 1982–1991 were obtained from the records maintained by midwives at the time of delivery. Data were provided by trained healthcare professionals^12^.

Data on VTE events and deaths were collected from the National Swedish Patient Register (NPR) and Cause of Death Registries. The NPR includes data on inpatient hospital care (85% complete 1980–1986, nationwide coverage from 1987) and specialised hospital-based outpatient clinics (since 2001) but primary care visits are not included^13^. The Cause of Death Registry includes the date and cause of death for all citizens of Sweden.

### Exposure variables and outcomes

Data on the participants’ educational level were obtained from the longitudinal integrated database for health insurance and labour market studies. Educational level was stratified into ≤ 9 years (the compulsory length of education in Sweden), 10–12 years and > 12 years (university or college). Smoking was stratified into 0, 1–9 and ≥ 10 cigarettes per day. Hypertension, congenital heart disease, and diabetes were defined as per the International Classification of Diseases, versions 8, 9 and 10. The prevalence of hypertension and diabetes at baseline was defined using the ICD-code in the NPR and/or self-reported diabetes/hypertension during their first antenatal visit to the MBR.

BMI at the first antenatal visit, the main exposure for the present analyses, was calculated by dividing body weight in kilograms by the square of height in metres (kg/m^2^). Participants were categorised into seven subgroups based on their BMI: 15– < 18.5 kg/m^2^, underweight; 18.5– < 20, normal weight (subcategorized into 20– < 22.5 and 22.5– < 25 kg/m^2^); overweight (subcategorized into 25– < 27.5 and 27.5– < 30 kg/m^2^); and obese (30– < 35 kg/m^2^) and severely obese (35– < 55 kg/m^2^) categories.

Primary and secondary diagnoses are registered in the NPR according to ICD-code (ICD-10 from 1997 and onwards; ICD-9, 1987–1996; and ICD8, 1968–1986). Pulmonary embolism and DVT were defined as a hospital discharge diagnosis or a cause of death according to ICD-8: PE (450), DVT (451); ICD-9: PE (415B, 416W) and DVT (451 except 451A); or ICD-10: PE (I26) and DVT (I80 except 180.0). Individuals were followed from the date of their first antenatal visit until the first diagnosis of PE, DVT, death or until the end of the study in December 31, 2014.

### Statistical analyses

The Cox proportional hazards regression models were used to estimate the associations between BMI at the first antenatal visit and VTE risk (PE and DVT) during follow-up, adding potential confounders to the models. Hazard ratios (HRs) and 95% confidence intervals (CIs) were calculated using low normal (20–22.5 kg/m^2^) BMI as reference values for all analyses. In a separate analysis, women who died of cancer were censored 2 years before death, postulating that malignancy may be a probable cause of VTE in these cases.

The follow-up period started 1 year after the date of the first antenatal visit, and all women were followed until the occurrence of PE, DVT or VTE, death or the end of follow-up (December 31, 2014), whichever occurred first. Only women without a PE or DVT prior to baseline were included in the study.

The proportional hazards assumptions were examined using tests based on weighted residuals^14^. Variables that did not fulfil the assumptions were stratified in the model. The baseline model was adjusted for age, parity and year of pregnancy. The second model was additionally adjusted for hypertension and diabetes, whereas the third model was further adjusted for smoking status and educational level. In the third model analysis, some data were missing, as shown in Table 1. Crude incidence rates and corresponding 95% CIs were calculated for outcomes separately as events per 100,000 person-years using Poisson regression. Baseline characteristics are provided as percentages or as means and standard deviation for continuous variables. All statistical modelling and analyses were performed using R version 3.6.2.

**Table 1:** Baseline characteristics of the study population by body mass index.

Variable	All	15 ≤ BMI < 18.5	18.50 ≤ BMI < 20	20 ≤ BMI < 22.5	22.5 ≤ BMI < 25	25 ≤ BMI < 27.5	27.5 ≤ BMI < 30	30 ≤ BMI < 35	35 ≤ BMI < 60	*p*
n	1,068, 040	43,382	118,523	344,880	276,112	140,964	68,412	55,683	20,084	
Age, years (SD)	27.5 ± 4.9	25.6 (4.7)	26.7 (4.7)	27.4 (4.8)	27.8 (4.9)	27.9 (5.0)	27.8 (5.2)	27.8 (5.2)	27.9 (5.1)	< 0.001
Height, mean (SD)	166.4 ± 6.3	166.1 (6.4)	166.6 (6.2)	166.7 (6.2)	166.5 (6.2)	166.2 (6.3)	166.0 (6.3)	165.9 (6.3)	166.1 (6.5)	< 0.001
Weight, mean (SD)	65.1 ± 12.0	48.8 (4.3)	53.7 (4.2)	59.1 (4.8)	65.5 (5.3)	72.1 (5.8)	78.8 (6.3)	88.0 (7.8)	106.2 (12.3)	< 0.001
Hypertension, n (%)	1050 (0.1)	24 (0.1)	83 (0.1)	251 (0.1)	255 (0.1)	161 (0.1)	113 (0.2)	114 (0.2)	49 (0.2)	< 0.001
Diabetes, n (%)	5092 (0.5)	52 (0.1)	197 (0.2)	1052 (0.3)	1501 (0.5)	1016 (0.7)	566 (0.8)	489 (0.9)	219 (1.1)	< 0.001
Smoking n (%), cigarettes/day										
0	899 842 (86.2)	32 583 (77.7)	95 741 (83.2)	291 469 (86.6)	237 447 (87.8)	120 858 (87.4)	58 019 (86.3)	46 915 (85.6)	16 810 (84.8)	< 0.001
1–9	101 640 (9.7)	6358 (15.2)	13 499 (11.7)	318 44 (9.5)	23 252 (8.6)	12 359 (8.9)	6481 (9.6)	5668 (10.3)	2179 (11.0)	< 0.001
≥ 10	42 433 (4.1)	2995 (7.1)	5818 (5.1)	13 259 (3.9)	9596 (3.6)	5014 (3.6)	2696 (4.0)	2219 (4.0)	836 (4.2)	< 0.001
Education, n (%)										
≤ 9 years	128 351 (12.1)	8693 (20.1)	16 024 (13.6)	38 035 (11.1)	29 513 (10.7)	16 480 (11.7)	8920 (13.1)	7714 (13.9)	2972 (14.8)	< 0.001
10–12 years	428 921 (40.2)	13 102 (30.3)	46 570 (39.4)	149 259 (43.4)	117 765 (42.8)	54 830 (39.0)	24 070 (35.3)	17 503 (31.5)	5522 (27.6)	< 0.001
> 12 years	501 388 (47.1)	20 960 (48.5)	54 601 (46.2)	154 506 (44.9)	126 617 (46.0)	68 484 (48.7)	34 804 (51.0)	29 998 (54.0)	11 418 (57.0)	< 0.001
NA	6792 (0.6)	457 (1.1)	927 (0.8)	2100 (0.6)	1539 (0.6)	849 (0.6)	444 (0.7)	353 (0.6)	123 (0.6)	< 0.001

*BMI* body mass index (kg/m^2^), *SD* standard deviation, *NA* not available (missing), *GUCH* grown-up congenital heart disease.

## Results

### Study population

Of the 1,393,346 women included in the study, 449 were excluded as their reported height was < 140 cm or > 200 cm; 1851 because of prior DVT, 741 because of prior PE, 759 because of a prior stroke at baseline (Fig. 1). Furthermore, 1435 women with a BMI of > 60 kg/m^2^ or < 15 kg/m^2^ were excluded. A total of 299,876 women were also excluded as it was not their first pregnancy, 21 women were excluded as they had inconclusive vital data, and 20,174 women was excluded as they had less than 1 year of follow-up time. Finally, the study population included 1,068,040 women. The mean BMI and age of the population were 23.5 kg/m^2^ and 27.5 years, respectively. Baseline information on age, height, weight, hypertension, diabetes, smoking status and educational level is shown in Table 1. The median follow-up time was 12.9 (interquartile range, 5.9, 21.2) years.

### Event rate

During follow-up, 3997 first VTE events were recorded (Table 2). The incidence rate for VTE per 100,000 person-years was 45.7 in women with a BMI of 30–34.9 kg/m^2^ and 61.1 with a BMI of 35–59.9 kg/m^2^ compared with 22.1 in women with a BMI of 20–22.4 kg/m^2^. Women fulfilling the obesity criteria were younger than those with normal weight at the time of VTE diagnosis: the mean age was 35.8 years in women with BMI of 30–35 kg/m^2^, 35.7 in BMI of 35–59.9 kg/m^2^ compared with 41.0 years in those with BMI of 20–22.4 kg/m^2^. Differences in the incidence rate and age at diagnosis for PE and DVT persisted when censoring women who died from cancer (Table 2).

**Table 2:** Events, event rates and age at diagnosis by BMI.

Variable	All	15 ≤ BMI < 18.5	18.5 ≤ BMI < 20	20 ≤ BMI < 22.5	22.5 ≤ BMI < 25	25 ≤ BMI < 27.5	27.5 ≤ BMI < 30	30 ≤ BMI < 35	35 ≤ BMI < 60	*p*
n	1,068,040	43,382	118,523	344,880	276,112	140,964	68,412	55,683	20,084	
Median follow up, years (IQR)	12.9 (5.9–21.2)	19.6 (8.6–27.3)	17.5 (7.7–26.4)	14.7 (6.7–24.6)	12.3 (5.8–20.2)	11.1 (5.2–18.4)	10.1 (4.7–17.2)	9.0 (4.1–15.5)	7.7 (3.7–12.9)	< 0.001
Venous thromboembolism (VTE)										
Crude events	3997	181	413	1166	1002	558	305	264	108	< 0.001
Event rate per 100,000 years (95% CI)	26.5 (25.7–27.3)	23.1 (19.9–26.7)	20.6 (18.7–22.7)	22.1 (20.9–23.4)	26.8 (25.2–28.5)	32.1 (28.5–34.9)	38.8 (34.5–43.4)	45.7 (40.3–51.5)	61.1 (50.2–73.8)	< 0.001
Age at diagnosis, years	40.1 ± 9.5	40.1 ± 9.7	41.1 ± 9.8	41.0 ± 9.4	40.3 ± 9.6	38.9 ± 9.2	40.4 ± 9.8	37.8 ± 9.0	35.7 ± 7.5	< 0.001
Pulmonary embolism (PE)										
Crude Events	2549	108	265	755	641	341	204	166	69	< 0.001
Event rate per 100,000 years (95% CI)	16.9 (16.2–17.6)	13.8 (11.3–16.6)	13.2 (11.7–14.9)	14.3 (13.3–15.4)	17.1 (15.8–18.5)	19.6 (17.6–21.8)	25.9 (22.5–29.7)	28.7 (24.5–33.4)	39.0 (30.3–49.4)	< 0.001
Age at diagnosis, years ± SD	40.9 ± 9.7	41.5 ± 10.3	41.8 ± 9.9	41.8 ± 9.4	40.9 ± 10.1	40.5 ± 9.1	41.1 ± 9.9	38.3 ± 9.0	34.7 ± 7.8	< 0.001
Deep venous thrombosis (DVT)										
Crude events	1765	83	179	507	435	261	126	126	48	< 0.001
Event rate per 100,000 years (95% CI)	11.7 (11.2–12.3)	10.6 (8.4–13.1)	8.9 (7.7–10.3)	9.6 (8.8–10.5)	11.6 (10.6–12.8)	15.0 (13.2–17.0)	16.0 (13.3–19.0)	21.8 (18.1–25.9)	27.1 (20.0–36.0)	< 0.001
Age at diagnosis, years ± SD	39.4 ± 9.2	38.9 ± 9.0	40.7 ± 9.6	39.9 ± 9.2	40.0 ± 9.3	37.5 ± 9.2	40.1 ± 9.8	37.5 ± 8.8	37.3 ± 7.0	< 0.001
PE excluding cancer death										
Crude events	2314	97	228	672	597	310	188	157	65	< 0.001
Event rate per 100,000 years (95% CI)	15.3 (14.7–16.0)	12.4 (10.0–15.1)	11.4 (9.9–13.0)	12.7 (11.8–13.7)	16.0 (14.7–17.3)	17.8 (15.9–19.9)	23.9 (20.6–27.5)	27.1 (23.1–31.7)	36.7 (28.4–46.8)	< 0.001
Age at diagnosis, years ± SD	40.2 ± 9.6	40.5 ± 10.2	40.7 ± 9.8	41.0 ± 9.2	40.3 ± 10.0	40.1 ± 9.1	40.8 ± 10.0	38.1 ± 9.1	34.3 ± 7.5	< 0.001
DVT excluding cancer death										
Crude events	1604	77	152	457	398	239	119	116	46	< 0.001
Event rate per 100,000 years (95% CI)	10.6 (10.1–11.2)	9.8 (7.7–12.3)	7.6 (6.4–8.9)	8.7 (7.9–9.5)	10.6 (9.6–11.7)	13.8 (12.1–15.6)	15.1 (12.5–18.1)	20.1 (16.6–24.1)	26.0 (19.0–34.7)	< 0.001
Age at diagnosis, years ± SD	38.8 ± 9.1	38.2 ± 8.8	39.6 ± 9.6	39.3 ± 9.1	39.5 ± 9.2	36.7 ± 8.8	39.7 ± 9.9	37.3 ± 8.9	36.9 ± 6.8	< 0.001

Data presented as mean ± SD, except for event rate (events/100 000) years (95% CI).

*IQR* interquartile range, *CI* confidence interval.

**Figure 2:**
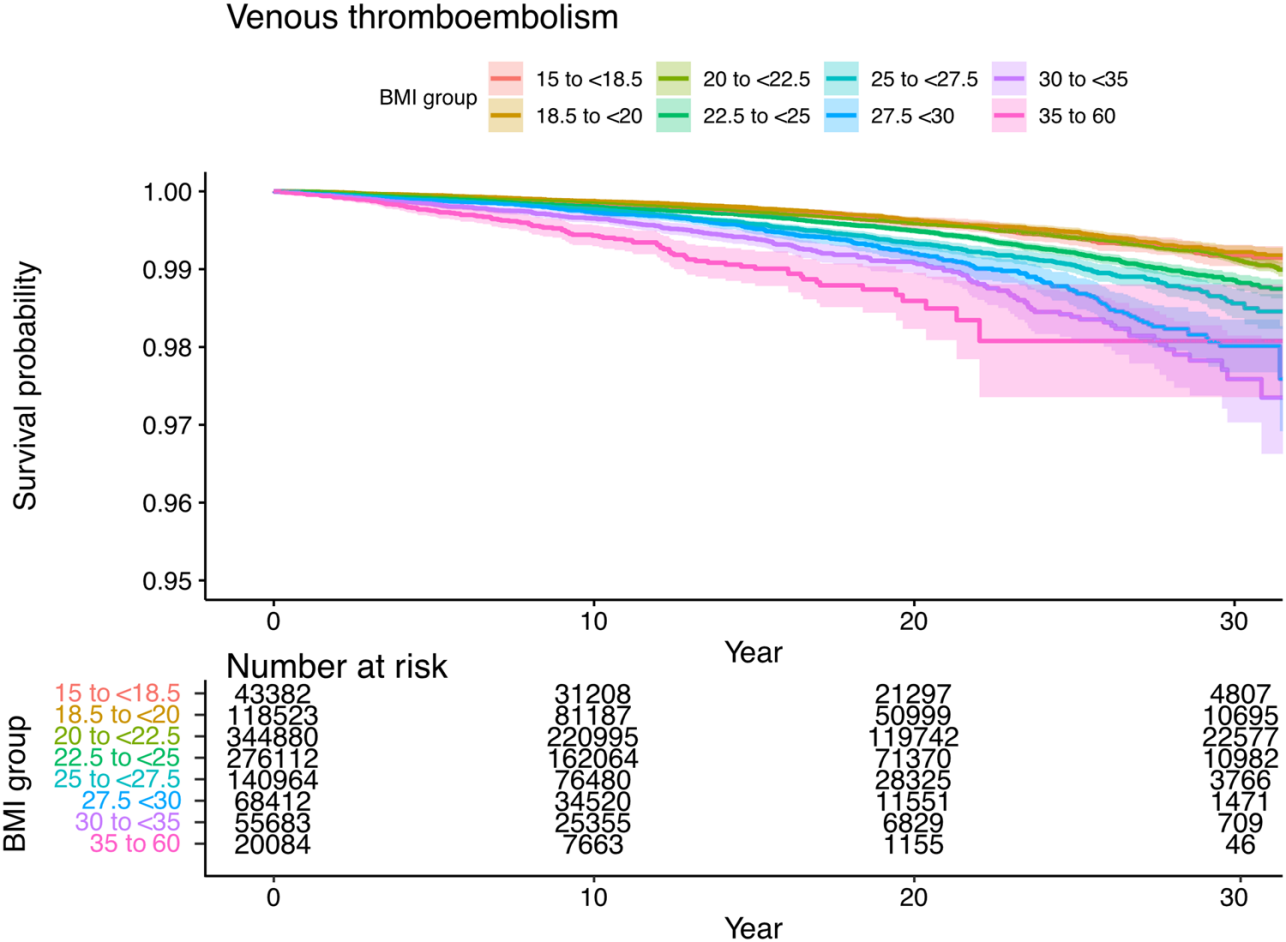
Kaplan–Meier plot for venous thromboembolism by BMI. Survival plot for VTE related to BMI. Survival probability is shown in the Y-axis and time in years in the X-axis.

Figure 2 shows a Kaplan–Meier plot for venous thromboembolism by BMI. Survival plot for VTE related to BMI. Survival probability is shown in the Y-axis and time in years in the X-axis. The survival curve showed an exponential increase in risk of VTE with longer follow-up (Fig. 2). Survival probability curves for DVT and PE as separate events can be found in supplementary materials (see Figs. S1 and S2).

### Hazard ratios

Women with high normal weight, BMI of 22.5–25.0 kg/m^2^, had an adjusted HR of 1.30 (95% CI 1.19–1.41) for VTE compared with women with BMI of 20–22.5 kg/m^2^ (Table 3) after adjusting for age, year of pregnancy, hypertension, diabetes, educational level, parity and smoking status. Corresponding HRs for women with BMI of 30–35 kg/m^2^ and 35–59.9 kg/m^2^ were 2.35 (95% CI 2.04–2.70) and 3.47 (95% CI 2.82–4.25). For DVT, the adjusted HR for women with BMI of 30–34.9 kg/m^2^ was 2.78 (95% CI 2.26–3.41) and for BMI 35–59.9 kg/m^2^, 4.08 (95% CI 3.00– 5.55). For PE, the adjusted HR for women with BMI of 30–34.9 kg/m^2^ was 2.20 (95% CI 1.85–2.62) and for BMI of 35–59.9 kg/m^2^, 3.20 (95% CI 2.48–4.14) compared with women with BMI of 20–22.4 kg/m^2^ (Table 3).

**Table 3:** Hazard ratios for VTE, DVT and PE by BMI group.

	Hazards ratio (95% CI)		
	Model 1	Model 2	Model 3
Venous thromboembolism, VTE			
15 ≤ BMI < 18.5	0.96 (0.82–1.12)	0.96 (0.82–1.12)	0.92 (0.78–1.08)
18.5 ≤ BMI < 20	0.89 (0.79–0.99)	0.89 (0.79–0.99)	0.88 (0.79–0.99)
20 ≤ BMI < 22.5	1 (Reference)	1 (Reference)	1 (Reference)
22.5 ≤ BMI < 25	1.30 (1.19–1.41)	1.29 (1.19–1.41)	1.30 (1.19–1.41)
25 ≤ BMI < 27.5	1.63 (1.47–1.80)	1.62 (1.46–1.80)	1.59 (1.43–1.76)
27.5 ≤ BMI < 30	2.01 (1.77–2.29)	2.00 (1.76–2.27)	1.95 (1.71–2.22)
30 ≤ BMI < 35	2.50 (2.18–2.86)	2.48 (2.16–2.84)	2.35 (2.04–2.70)
35 ≤ BMI < 60	3.74 (3.06–4.57)	3.70 (3.03–4.53)	3.47 (2.82–4.25)
Deep vein thrombosis, DVT			
15 ≤ BMI < 18.5	0.98 (0.78–1.24)	0.98 (0.78–1.24)	0.98 (0.77–1.24)
18.5 ≤ BMI < 20	0.87 (0.73–1.03)	0.87 (0.73–1.03)	0.86 (0.72–1.03)
20 ≤ BMI < 22.5	1 (Reference)	1 (Reference)	1 (Reference)
22.5 ≤ BMI < 25	1.32 (1.16–1.51)	1.32 (1.16–1.50)	1.31 (1.14–1.49)
25 ≤ BMI < 27.5	1.82 (1.57–2.12)	1.82 (1.56–2.11)	1.78 (1.52–2.08)
27.5 ≤ BMI < 30	2.01 (1.65–2.45)	2.00 (1.64–2.44)	2.01 (1.64–2.45)
30 ≤ BMI < 35	2.96 (2.43–3.62)	2.93 (2.40–3.57)	2.78 (2.26–3.41)
35 ≤ BMI < 60	4.34 (3.21–5.87)	4.29 (3.17–5.80)	4.08 (3.00–5.55)
PE excluding cancer death *α*			
15 ≤ BMI < 18.5	0.89 (0.73–1.09)	0.89 (0.73–1.09)	0.84 (0.68–1.03)
18.5 ≤ BMI < 20	0.88 (0.77–1.02)	0.88 (0.77–1.02)	0.88 (0.76–1.02)
20 ≤ BMI < 22.5	1 (Reference)	1 (Reference)	1 (Reference)
22.5 ≤ BMI < 25	1.27 (1.14–1.41)	1.27 (1.14–1.41)	1.28 (1.14–1.42)
25 ≤ BMI < 27.5	1.50 (1.32–1.71)	1.50 (1.32–1.71)	1.47 (1.29–1.68)
27.5 ≤ BMI < 30	2.03 (1.73–2.37)	2.02 (1.73–2.36)	1.94 (1.65–2.28)
30 ≤ BMI < 35	2.34 (1.97–2.78)	2.33 (1.96–2.76)	2.20 (1.85–2.62)
35 ≤ BMI < 60	3.47 (2.70–4.46)	3.46 (2.69–4.45)	3.20 (2.48–4.14)
DVT excluding cancer death β			
15 ≤ BMI < 18.5	1.00 (0.79–1.28)	1.00 (0.79–1.28)	0.99 (0.77–1.27)
18.5 ≤ BMI < 20	0.82 (0.68–0.98)	0.82 (0.68–0.98)	0.80 (0.66–0.97)
20 ≤ BMI < 22.5	1 (Reference)	1 (Reference)	1 (Reference)
22.5 ≤ BMI < 25	1.34 (1.17–1.53)	1.33 (1.17–1.53)	1.32 (1.15–1.51)
25 ≤ BMI < 27.5	1.83 (1.56–2.14)	1.82 (1.56–2.14)	1.80 (1.53–2.12)
27.5 ≤ BMI < 30	2.08 (1.70–2.55)	2.07 (1.69–2.53)	2.08 (1.69–2.55)
30 ≤ BMI < 35	2.97 (2.42–3.66)	2.93 (2.39–3.61)	2.79 (2.26–3.46)
35 ≤ BMI < 60	4.50 (3.30–6.13)	4.44 (3.26–6.05)	4.22 (3.08–5.78)
Pulmonary embolism, PE			
15 ≤ BMI < 18.5	0.90 (0.73–1.11)	0.90 (0.73–1.11)	0.83 (0.67–1.04)
18.5 ≤ BMI < 20	0.86 (0.74–1.00)	0.86 (0.74–1.00)	0.86 (0.74–1.00)
20 ≤ BMI < 22.5	1 (Reference)	1 (Reference)	1 (Reference)
22.5 ≤ BMI < 25	1.32 (1.18–1.47)	1.31 (1.18–1.47)	1.32 (1.18–1.47)
25 ≤ BMI < 27.5	1.51 (1.32–1.73)	1.51 (1.32–1.73)	1.47 (1.28–1.69)
27.5 ≤ BMI < 30	2.06 (1.75–2.42)	2.05 (1.74–2.41)	1.95 (1.65–2.30)
30 ≤ BMI < 35	2.42 (2.03–2.89)	2.40 (2.01–2.87)	2.27 (1.89–2.72)
35 ≤ BMI < 60	3.53 (2.73–4.58)	3.52 (2.72–4.56)	3.23 (2.48–4.21)

Reference group is BMI 20–22.5. PE and DVT are adjusted for cancer death within 2 years prior to VTE. Associations adjusted for groups of covariates called Model 1, 2 and 3.

In a separate analysis, censoring women who died from cancer within 2 years, women with a BMI of 30–34.9 kg/m^2^ had a HR of 2.20 (95% CI 1.85–2.62) for PE compared with women with a BMI of 20–22.49 kg/m^2^. The corresponding HR for BMI of 35–59.9 kg/m^2^ was 3.20 (95% CI 2.48–4.14). For DVT, women with BMI of 30–34.9 kg/m^2^ had an HR of 2.27 (95% CI 1.89–2.72) and women with BMI of 35–59.9 kg/m^2^ 3.23(95% CI 2.48–4.21) compared with women with BMI of 20–22.49 kg/m^2^.

## Discussion

In this large-scale registry-based cohort study, we found a strong, near-linear association between BMI and long-term post-pregnancy risk of VTE, which was already evident at mildly elevated body weight in early pregnancy. Almost a fourfold increase in long-term risk of VTE was found in the severely obese group relative to low normal weight, which is in agreement with other studies in older subjects^4, 9, 15^; however, adding that the VTE risk associated with obesity among younger women seems to be higher than previously shown.

The study results are comparable to the results of two previous studies on young men^11, 16^. Conversely, an American population-based, nested case–control study did not identify an elevated BMI as a risk factor for VTE^17^. However, that study only included 625 participants with several missing data on body weight and height. Compared with findings from the large Emerging Risk Factor Collaboration^10^ in mainly middle-aged persons, and with a lower number of VTE events than in our study, HRs associated with obesity and severe obesity were lower than in our study. No previous study has assessed long-term VTE risk associated with weight in younger women with a similarly large number of events.

All women included in the study were pregnant at baseline; however, the baseline weight refers to early pregnancy (weeks 8–12), with only marginal weight gain resulting from pregnancy^18^. However, one problem with only including pregnant women is that they might be healthier than the average woman.

The mechanisms underlying the increased risk of VTE in obesity are not fully understood. A study conducted in 2016 suggested that body weight relates to VTE because of physical factors associated with the blood flow and not the inflammation or hypercoagulability that was proposed to be associated with adiposity^19^. Conversely, another study found that adipose tissue could play a role in the pro-thrombotic state observed in obesity by affecting coagulation, haemostasis and fibrinolysis^20^. Moreover, it should be emphasised that the increased VTE risk mediated by overweight and obesity early in life can be reversed by weight reduction^21^.

The adjustments considered for the models in this study only marginally affected HRs. In sensitivity analyses, we explored the effects of excluding strongly provoked DVT and PE cases (cancer death within 2 years after VTE). These exclusions did not affect the results.

### Strengths and limitations

The strengths of this study include the large study population and the near-complete coverage. Another strength is the long-term prospective follow-up. However, it should be noted that the coverage of the inpatient registry was not yet nationwide until 1987, which means that some early VTE events may not have been recorded. Conversely, patients treated on an outpatient basis during the last years of follow-up will not have been included. In addition, the MBR only includes women who gave birth to live babies and those who had pregnancy losses and stillborn children (only 3.5–4 per thousand of all pregnancies in Sweden^22^ after the third trimester. Consequently, women with pregnancy loss before the third trimester, and women who are voluntarily or involuntarily childless is not included and results from this study may not reflect VTE risk among these women. Involuntary childlessness is more common among women with obesity^23^, so it is possible that the incidence of VTE may have been underestimated. In addition, we did not take into account subsequent pregnancies during follow-up nor the increased risk each pregnancy entails. However, as about 85% of women in Sweden give birth, with the average number of live births per woman in Sweden below two, it is unlikely that a selective effect of BMI on a first or any further pregnancies will have affected our results.

Other potential limitations include the lack of information on subsequent BMI during follow-up. Given the strong tracking of body weight over lifetime and the likelihood of weight gain during adulthood, it is likely that many women in the normal weight group will have progressed into the overweight or obesity groups later on^24^. Once established, overweight and obesity are difficult to treat. The extent to which weight loss reduces the excess VTE risk could not be established in the present study.

Another limitation is that the diagnoses were not verified. However, in a Swedish study including nearly 400 VTE cases, where hospital records were retrieved for approximately 80%, the diagnosis was incorrect in only 13 cases (which were excluded) and objectively verified in almost all remaining cases^25^. Another Swedish cohort study identified 2450 participants with a first-time diagnosis of PE or DVT in the Swedish patient registry^26^. While the positive predictive value for a diagnosis of PE was 81% for PE, it was only 59% for a DVT. This difference was thought to be due to patients with DVT in a larger proportion being treated as outpatients. Still, findings with respect to BMI and VTE were similar for PE and DVT, and in our analyses we did not include patients treated on an outpatient basis. Further, if misclassification occurs, it may not be random, with obese women potentially underdiagnosed, as well as overdiagnosed with PE or DVT.

Other intervening factors that might have affected the relation between BMI and VTE is, for example, hospitalizations, or non-fatal cancers, both potentially more common among obese women, but also rare among these young and predominantly healthy individuals. In ongoing analyses, we found associations between BMI and cancer to be complex and varying by cancer type, and a similar complexity will likely also apply to hospitalizations.

The effects of hormonal treatment, either for contraception or as hormone replacement therapy are well known^27^; however, potential interactions with obesity remain to be explored^28^. Furthermore, postmenopausal women who use oestrogen have an increased VTE risk, at least during the first year of treatment^29^, but this information was not available.

In conclusion, we found that overweight or obese women were at markedly high risk of developing VTE later in life compared with women with normal weight, with risk starting to increase already at normal BMI levels. An important clinical implication of this study is that overweight and obesity are important risk factors for later VTE development among young pregnant women. Women with obesity, particularly those with severe obesity, had a markedly increased risk. Given that obesity and overweight are increasing worldwide, this adds to the numerous reasons to maintain low normal weight throughout adulthood to reduce the risk of VTE and other adverse health outcomes.

### Supplementary Information

Supplementary Information.

## Data Availability

The data that support the findings of this study are available from the Swedish Medical Birth Registry, the Swedish Inpatient and Outpatient Registry, and the Swedish Cause of Death Registry, held by the Swedish National Board of Health and Welfare, and the LISA registry held by Statistics Sweden. Researchers can apply for these data by contacting these government agencies, fulfilling legal and regulatory requirements, and providing an acceptance letter from the Swedish Ethical Review Authority. For legal reasons, these datasets are not directly available from the corresponding author.
