# Supplementary material for: Prepregnancy overweight and obesity and long-term risk of venous thromboembolism in women
Source: Sci Rep. 2023 Sep 5;13:14597. doi: 10.1038/s41598-023-41186-2 (PMC10480468; doi:10.1038/s41598-023-41186-2)

**Supplementary Figure S1.** Kaplan-Meier curves for deep vein thrombosis by BMI. Survival probability is shown on the Y-axis and time in years on the X-axis. Dfds,mdsmg,dfmg klfdmgklmfgkmfgkmf

**
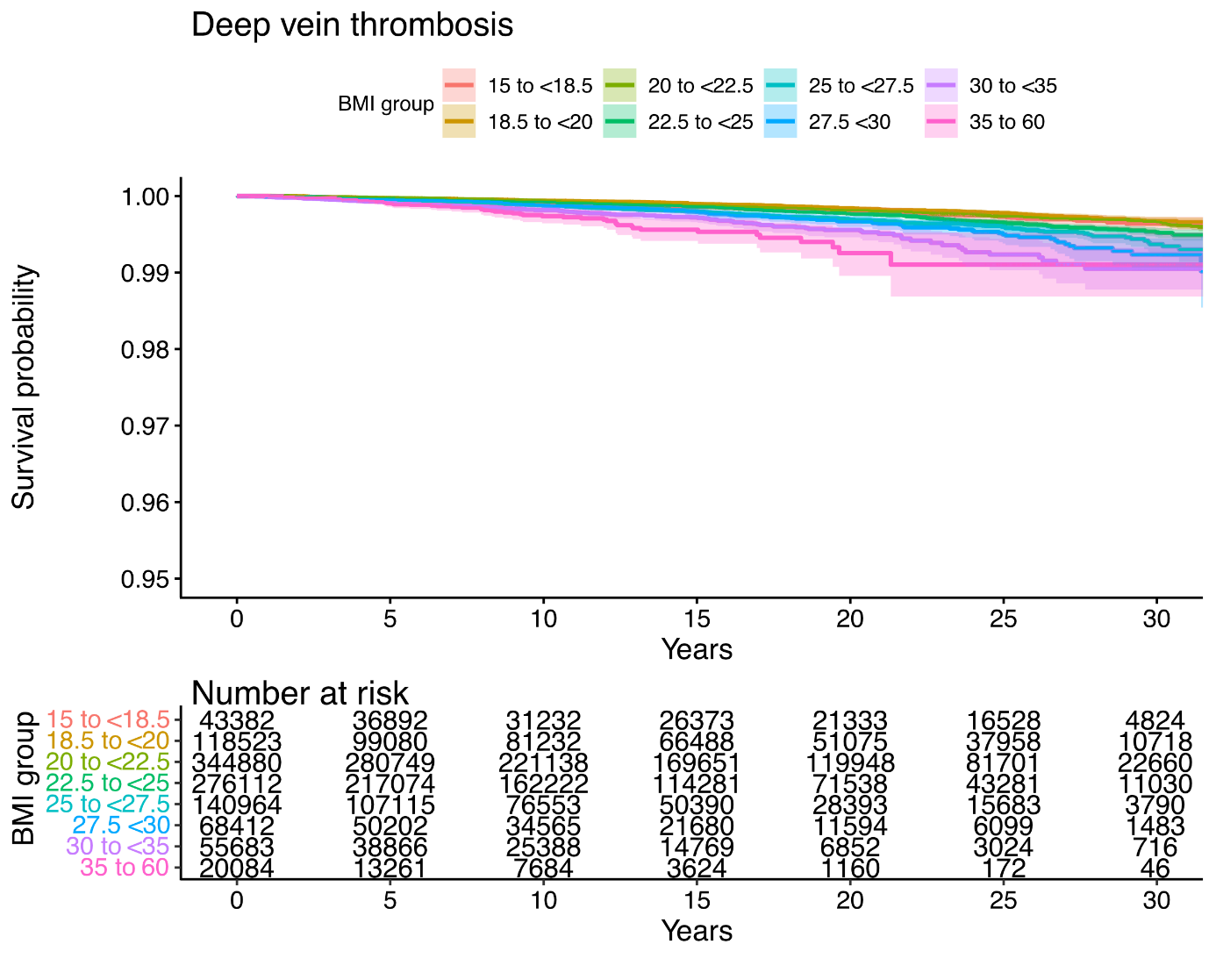
**

**Supplementary Figure 2.** Kaplan-Meier curves for pulmonary embolism by BMI. Survival probability is shown on the Y-axis and time in years on the X-axis. rvival probability is shown in the Y-axis and time in years in the X-axis.


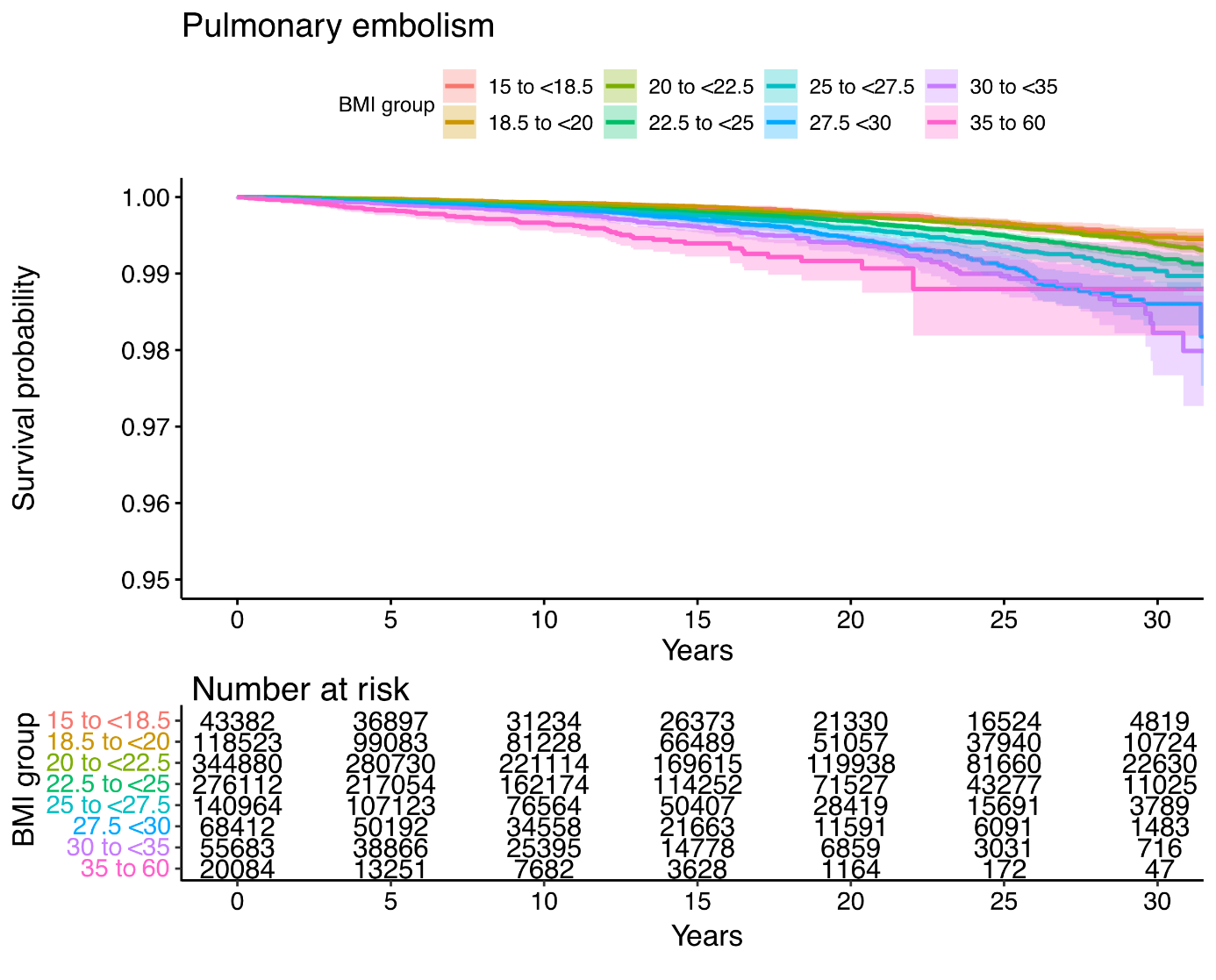

Supplement: Supplementary file 1 — Supplementary Information. [file 41598_2023_41186_MOESM1_ESM.docx]
